# The time to act is now if we are to reduce the impact of climate change on global health

**DOI:** 10.5588/pha.24.0047

**Published:** 2024-12-01

**Authors:** T. Scirocco, K.U. Eman, M.C. Raviglione, G.N. Kazi

**Affiliations:** ^1^ Centre for Multidisciplinary Research in Health Science (MACH), University of Milan, Italy;; ^2^ Dopasi Foundation, Islamabad, Pakistan.

**Keywords:** Sustainable Development Goals, SDGs, poverty, food security, water security, mental health

Once regarded primarily as an environmental challenge, climate change has emerged as a significant threat to global health.^1–3^ Its effects are insidious, far-reaching, and interconnected with nearly every facet of human health. Climate change attacks the very foundations of human health by disrupting environmental and social determinants and the sources of life: clean water to drink, sufficient food to eat, secure shelter and clean air to breathe. Rapid action is essential to build resilience, focusing on solutions that encompass health, human rights and economic benefits.^4^ As global temperatures rise, we face increased frequency of extreme weather events, shifting patterns of infectious disease, and water- and vector-borne diseases. For vulnerable populations, including the elderly, children, women and those with preexisting health conditions, the risks are higher. Likewise, populations living on small islands and coastal regions or within megacities, mountainous or polar regions, are exposed to the consequences of global warming.^1,5^ Public health professionals must recognise that climate change is a significant threat to human health, jeopardising food and water security – and not just a threat for the future, but happening in front of our eyes as its impact grows each year. Yet, public awareness and politically sound engagement remain inadequate, especially in low- and middle-income countries (LMICs) where these effects are most severe.

Despite global commitments, significant barriers persist, including time constraints, limited capacity, and difficulty integrating climate considerations into already strained health systems.^1^ Global health professionals, particularly in the public health sector, must build resilience within health systems to protect the most vulnerable populations from the escalating health impacts. Central to this effort is reducing carbon emissions through a global shift to renewable energy sources, improved energy efficiency and more sustainable public transport. However, there is also a pressing need to reinforce the capacity of health systems.^3^ Furthermore, although the United Nations established Sustainable Development Goals (SDGs) seek to promote sustainability in food production, supply and consumption, if the ecological footprint of the production chain is not managed effectively, these can also jeopardise the Earth's biosphere and, consequently, human health.^6^

Since 2008, the WHO has played a leading role in encouraging dialogue about the health impacts of climate change and issued a series of guidance documents to help national governments recognise and respond to these growing threats.^7–10^ However, despite these recommendations, there has been little evidence of their implementation, particularly in LMICs where the gap between policy and action is most glaring.^11^ In these regions, it is impossible to overstate the need for increased urgency to increase funding, design health interventions and evaluation mechanisms. We have advocated for governments to be accountable for their actions,^12,13^ starting with reliable and standardised reporting to understand the health impacts of climate change and shape evidence-based policies that protect their communities. With a focused and multidisciplinary effort – and a One Health and planetary health perspective – we can prevent worsening outcomes for public health.

## Climate change and disease transmission

As the climate crisis deepens, more people will be displaced by natural disasters and environmental degradation (such as drought and desertification, which reduce agricultural productivity). This will lead to further conflict, food and water insecurity, and changes in disease transmission. Climate-sensitive diseases, such as malaria, dengue and leishmaniasis, are spreading to previously unaffected areas, adding to existing health challenges. Addressing the migration-health nexus requires frameworks that clarify how human mobility affects climate and health outcomes.^14^ Another example is TB, already the leading infectious killer globally and a major challenge in LMICs – climate change will likely make it even harder to manage. Factors such as poverty, undernutrition, overcrowding and poor indoor air quality (already risk factors for TB) are worsened by climate-related pressures. Research increasingly links worsening environmental conditions to heightened TB susceptibility, as it exacerbates the underlying determinants. A systematic review of 53 studies revealed positive associations between climate change and TB risk factors (such as undernutrition, diabetes and indoor air pollution).^15^ Given we are already struggling to achieve the targets of the End TB strategy, and it is evident that climate change will make TB control an even greater challenge,^15^ public health leaders must quickly incorporate climate adaptation strategies into TB control programmes.

## Climate change and mental health

Although effects on physical health often garner the most attention, climate change's impact on mental health is also profound and far-reaching. Studies show that individuals who are acutely aware of climate change are more likely to experience increased levels of anxiety, depression and eco-anxiety, especially among younger populations who view the climate crisis as a direct threat to their future.^16^ A review of over 3,000 articles found strong associations between climate change awareness and a range of mental health issues, including stress, adjustment disorders and even suicidal thoughts. Integrating mental health care into climate adaptation and disaster response programmes will be critical as the global climate worsens.^17^

The evidence presented leads to a simple conclusion: the time to act is now. Climate change is already transforming the health landscape, and without swift and coordinated responses, we will continue to see worse health outcomes for millions of people. For public health professionals, this means integrating climate change considerations into every facet of health system planning and response. It requires collaboration with sectors outside of health (such as energy, housing, transport and agriculture) to build comprehensive, climate-resilient systems. The healthcare sector must also improve its sustainability efforts. While advances in artificial intelligence are transforming diagnostics and patient outcomes, the energy demands of these technologies also contribute to the carbon footprint of healthcare systems. Studies in various countries (e.g., Canada, Australia, the United Kingdom and the United States) have shown that healthcare may contribute 4–10% of greenhouse gas and pollutant emissions. Sustainable practices must, therefore, be prioritised – including for TB control^18^ – to ensure that the innovations we embrace today do not contribute to the very problems we are trying to solve.^19,20^

Climate change is reshaping global health in profound ways and exacerbating existing vulnerabilities in populations already struggling with poverty and disease. As public health activists, we must lead the way by advocating for increased climate resilience of health systems and engendering policies that place health at the centre of climate action. The future of global health depends on our ability to address this crisis head-on, with a sense of urgency and an unwavering commitment to human equity, financial sustainability and social justice.

## References

[1] Kotcher J, Views of health professionals on climate change and health: a multinational survey study. Lancet Planet Health. 2021;5(5):e316–323. 33838130 10.1016/S2542-5196(21)00053-X PMC8099728

[2] Campbell-Lendrum D, Climate change and health: three grand challenges. Nat Med. 2023;29(7):1631–1638. 37464036 10.1038/s41591-023-02438-w

[3] Deivanayagam TA, Osborne RE. Breaking free from tunnel vision for climate change and health. PLoS Glob Public Health. 2023;3(3):e0001684. 36963098 10.1371/journal.pgph.0001684 PMC10021701

[4] Ebi KL, Extreme weather and climate change: population health and health system implications. Annu Rev Public Health. 2021;42(1):293–315. 33406378 10.1146/annurev-publhealth-012420-105026 PMC9013542

[5] Kim KH, Kabir E, Ara Jahan S. A review of the consequences of global climate change on human health. J Environ Sci Health, Part C. 2014;32(3):299–318. 10.1080/10590501.2014.941279 25226222

[6] Bocchi S, Agrofood system and human health. In: Ingegnoli V, Lombardo F, La Torre G, eds. Environmental alteration leads to human disease: a planetary health approach. Cham, Switzerland: Springer, 2022: pp 131–163.

[7] World Health Organization. Climate change and health. Geneva, Switzerland: WHO, 2008.

[8] World Health Organization. Gender, climate change and health. Geneva, Switzerland: WHO, 2014.

[9] World Health Organization. WHO global strategy on health, environment and climate change. Geneva, Switzerland: WHO, 2020.

[10] World Health Organization. 2021 WHO health and climate change global survey report. Geneva, Switzerland: WHO, 2021.

[11] Scheelbeek PF, The effects on public health of climate change adaptation responses: a systematic review of evidence from low-and middle-income countries. Environmental Research Letters. 2021 Jul 13;16(7):073001. 34267795 10.1088/1748-9326/ac092c PMC8276060

[12] Kazi GN, Blackbourn HD. Multisectoral action and community involvement are required for long-term improvements in public health. Public Health Action. 2024;14(2):43–44. 38957500 10.5588/pha.24.0022 PMC11216293

[13] Eman KU, Sivapuram MS, Kazi GN. Community Connect: bridging the gap between scientific research and community engagement for TB elimination. Public Health Action. 2024;14(1):1. 38798780 10.5588/pha.24.0005 PMC11122708

[14] McMichael C. Human mobility, climate change, and health: Unpacking the connections. Lancet Planet Health. 2020;4(6):e217–218. 32559436 10.1016/S2542-5196(20)30125-X

[15] Kharwadkar S, The impact of climate change on the risk factors for tuberculosis: A systematic review. Environ Res. 2022;212:113436. 35550808 10.1016/j.envres.2022.113436

[16] Newberry Le Vay J, Integrating mental health into climate change education to inspire climate action while safeguarding mental health. Front Psychol. 2024;14:1298623. 38259528 10.3389/fpsyg.2023.1298623 PMC10800611

[17] Gianfredi V, Climate change perception and mental health. results from a systematic review of the literature. Eur J Investig Health Psychol Educ. 2024;14(1):215–229. 10.3390/ejihpe14010014 PMC10814599 38248134

[18] Harries AD, Martinez L, Chakaya JM. Tackling climate change: measuring the carbon footprint of preventing, diagnosing and treating TB. Public Health Action. 2021;11(1):40. 33777720 10.5588/pha.20.0076 PMC7987244

[19] Ueda D, Climate change and artificial intelligence in healthcare: Review and recommendations towards a sustainable future. Diagnostic and Interventional Imaging. 2024 Jun 24; doi: 10.1016/j.diii.2024.06.002 [Online ahead of Print]. 38918123

[20] Eckelman MJ, Sherman JD, MacNeill AJ. Life cycle environmental emissions and health damages from the Canadian healthcare system: an economic-environmental-epidemiological analysis. PLoS Medicine 2018;15(7):e1002623. 30063712 10.1371/journal.pmed.1002623 PMC6067712

